# Conditional survival among patients with adrenal cortical carcinoma determined using a national population-based surveillance, epidemiology, and end results registry

**DOI:** 10.18632/oncotarget.5831

**Published:** 2015-10-14

**Authors:** Wen-jun Xiao, Yao Zhu, Bo Dai, Hai-liang Zhang, Guo-hai Shi, Yi-jun Shen, Yi-ping Zhu, Ding-wei Ye

**Affiliations:** ^1^ Department of Urology, Fudan University Shanghai Cancer Centre, Shanghai 200032, People's Republic of China; ^2^ Department of Oncology, Shanghai Medical College, Fudan University, Shanghai 200032, People's Republic of China

**Keywords:** adrenal cortical carcinoma, conditional survival, overall survival, surgical excision

## Abstract

Surgical excision is essential for management of the rare and aggressive neoplasm adrenal cortical carcinoma (ACC). Five-year overall survival (OS) after surgery for ACC is dependent on disease stage, but for all stages the risk of death declines with time after surgery. We calculated the effect of post-surgical duration on conditional survival (CS) among ACC patients. A total of 641 patients with M0 ACC were selected from the Surveillance, Epidemiology, and End Results (SEER) registry (1988–2012). OS for the entire cohort at 1, 2, 3, 4, 5 and 6 years was 81.4%, 66.8%, 56.3%, 50.3%, 47.2% and 44.3%, respectively. CS for an additional year given prior survival for 0, 1, 2, 3, 4 or 5 years was 81.4%, 81.1%, 83.0%, 87.5%, 93.4% and 93.4%, respectively. Age, tumor stage, tumor grade and marital status affected OS and CS. Increases in 1-year CS over time were more pronounced in patients with poorer prognostic factors. With longer follow-up, tumor stage- and grade-dependent differences in CS decreased or even disappeared. CS may provide more meaningful life expectancy predictions for survivors of ACC than conventional survival analysis.

## INTRODUCTION

Adrenal cortical carcinoma (ACC) is a rare and aggressive neoplasm with a reported annual incidence of 0.7–2.0 cases per million [1, 2]. Most patients diagnosed with ACC present with advanced disease. For those with local or locally advanced disease, radical resection of the primary tumor is the only curative option [3–5]. Following curative resection, overall survival remains low, but the risk of dying of ACC is not constant over time, as most deaths occur within the first 2 years after the surgery. Consequently, prognosis (e.g., 5-year survival probability from the day of surgery) improves conditionally depending on the length of time beyond a critical preliminary period the patient has survived. This improvement in prognosis over time can be explained as a conditional probability of survival.

To our knowledge, there are no published studies examining conditional survival (CS) among patients with ACC. The rarity of ACC makes both prospective and retrospective single-institution or multi-institution studies difficult. The Surveillance, Epidemiology, and End Results (SEER) registry, though imperfect, is a valuable source of data on rare tumors, including ACC. For this study, we used recent data from the SEER registry on patients who had undergone surgery for ACC to calculate the conditional probability of survival. This enabled us to evaluate the utility of surgery for ACC from a new perspective.

## RESULTS

The clinical stage classifications of ACC are shown in Table 1. In all, 641 patients with nonmetastatic ACC diagnosed between 1988 and 2012 were identified in the SEER database. All of these patients had undergone surgery to remove an adrenal tumor. Their relevant sociological, clinical and pathological characteristics are summarized in Table 2. The median age at diagnosis was 53 (18–89) years, and there were more women (63%, *n* = 402) than men (37%, *n* = 239). According to the tumor-staging classification suggested by the European Network for the Study of Adrenal Tumours (ENSAT), most patients had stage II disease (47%), followed by stage III (33%) and stage I (5%). The ENSAT stages were unknown in 15% of patients. Those with unknown stages were diagnosed earlier (median year of diagnosis 1998; *P* < 0.001) than those classified as stage I/II or III (median years 2005, and 2006, respectively). Differences in age, sex, tumor size, race, laterality and marital status were not statistically significant among the stage groups. Patients with higher stage disease tended to have higher grade tumors, a greater rate of lymph node resection and radiation therapy, and were more likely to experience a cancer-specific death.

**Table 1:** Clinical stage of adrenal cortical carcinoma

TNM		UICC		ENSAT	
T1	≤5 cm, no extra-adrenal invasion	I	T1 N0 M0	I	T1 N0 M0
T2	>5 cm, no extra-adrenal invasion	II	T2 N0 M0	II	T2 N0 M0
T3	Local invasion	III	T1–2 N1 M0	III	T3–4 N0 M0
T4	Adjacent organs		T3 N0		T1–4 N1 M0
N1	Regional	IV	T3 N1	IV	Any T Any N M1
M1	Distant		T4 Any N		
			Any T Any N M1		

UICC = Union for International Cancer Control; ENSAT = European Network for the Study of Adrenal Tumours

**Table 2:** Relevant sociological, clinical and pathological characteristics of the entire cohort and patients segregated based on disease stage

	Stage I and II		Stage III		Stage Unknown		P*	Total	
	Median	Range	Median	Range	Median	Range		Median	Range
**Year of Diagnosis**	2005	1988–2012	2006	1988–2012	1998	1988–2012	<0.001	2004	1988–2012
**Age at Diagnosis (yr)**	53	18–89	54	20–87	52	18–88	0.286	53	18–89
**Size in mm**	100	12–800	117	12–280	105	1–990	0.157	105	1–990
**N and %**	332	52	215	33	94	15		641	
**Sex**							0.103		
Male	117	35	92	43	30	32		239	37
Female	215	65	123	57	64	68		402	63
**Race**							0.146		
White	279	84	192	89	84	89		555	87
Other	53	16	23	11	10	11		86	13
**Marital status**							0.282		
Divorced	23	7	17	8	9	10		49	8
Married	209	63	142	66	54	57		405	63
Single	70	21	37	17	18	19		125	20
Separated	1		3	1	0	0		4	1
Widowed	25	8	12	6	11	12		48	7
Unknown	4	1	4	2	2	2		10	2
**Laterality**							0.063		
Left	193	58	103	48	50	53		346	54
Right	139	42	108	50	44	47		291	45
Unknown or bilateral			4	2				4	1
**Tumor Grade**							0.026**		
I/II	38	11	18	8	13	14		69	11
III/IV	40	12	43	20	10	11		93	15
Unknown	254	77	154	72	71	76		479	75
**Lymph node examined**							<0.001		
No	273	82	127	59	88	94		488	76
Yes	54	16	83	39	2	2		139	22
Unknown	5	2	5	2	4	4		14	2
**Radiotherapy**							0.0067		
No or unknown	312	94	185	86	86	91		583	91
Yes	20	6	30	14	8	9		58	9
**Cause of death**							<0.001		
Alive	186	56	77	36	28	30		291	45
Dead (due to ACC)	110	33	122	57	51	54		283	44
Dead (due to other reasons)	36	11	16	7	15	16		67	10

* compared among stage I/II, stage III and stage Unknown groups using analysis of variance and χ2 test for continuous variables and for categorical variables, respectively.

** compared among stage I/II, stage III and stage unknown groups using Fisher's exact probability test. Cases with unknown grade were excluded.

The median overall survival (OS) among the entire cohort was 51 (43–70) months. One-year and 5-year OS rates were 81.4% and 47.2%, respectively. Univariate analysis showed that age, tumor stage, marital status and tumor grade had significant effects on OS (*p* < 0.01). These prognostic factors were also significant in a multivariate analysis (Table 3). For the subgroup with stage I/II disease, the median OS was 85 (61–163) months, with 1-year and 5-year OS rates of 87.0% and 56.1%, respectively. For the subgroup with stage III disease, the median OS was 25 (20–33) months, with 1-year and 5-year OS rates of 71.2% and 33.8%, respectively. For the subgroup with unknown stage disease, the median OS was 51 (33–88) months, with 1-year and 5-year OS rates of 84.2% and 47.3%, respectively. OS among the entire cohort at 1, 2, 3, 4, 5 and 6 years was 81.4%, 66.8%, 56.3%, 50.3%, 47.2% and 44.3%, respectively. As shown in Table 4, CS for an additional year after survival for 0, 1, 2, 3, 4 or 5 years was 81.4%, 81.1%, 83.0%, 87.5%, 93.4% and 93.4%, respectively.

**Table 3:** Prognostic factors affecting overall survival among adrenal cortical carcinoma patients

Variable at the time of diagnosis	Univariate Analysis	Multivariate Analysis	HR	95% CI
**Year of Diagnosis**	NS			
**Age at Diagnosis (yr)**				
≤53	Reference			
>53	<0.001	<0.001	1.521	1.228–1.885
**Sex**	NS			
**Race**	NS			
**Marital status**				
Married, Single, Unknown	Reference			
Divorced, Separated, Widowed	0.009	0.038	1.343	1.016–1.775
**Laterality**	NS			
**Size** (mm)	NS			
**Clinical stage**				
I/II	Reference			
III	< 0.001	< 0.001	1.932	1.527–2.446
Unknown	0.025	0.017	1.429	1.066–1.916
**Tumor Grade**				
I/II	Reference			
III/IV	< 0.001	0.005	1.966	1.226–3.152
Unknown	< 0.001	0.005	1.792	1.195–2.686
**Number of Lymph nodes examined**	NS (only for lymph node negative cases)			
**Radiotherapy**	NS			

**Table 4:** Conditional survival for an additional year among patients segregated based on prognostic factors

N year since diagnosis*	2nd	3rd	4th	5th	6th
**Entire cohort**	0.811	0.830	0.875	0.934	0.934
**Age at Diagnosis (yr)**	*P* = 0.037	*P* = 0.103	*P* = 0.234	*P* = 0.220	*P* = 0.044
≤53	0.835	0.840	0.880	0.916	0.935
>53	0.778	0.814	0.868	0.964	0.932
**Marital status**	*P* = 0.094	*P* = 0.050	*P* = 0.016	*P* = 0.009	*P* = 0.017
Married, Single, Unknown	0.816	0.831	0.884	0.939	0.949
Divorced, Separated, Widowed	0.781	0.823	0.820	0.899	0.814
**Clinical stage**	*P* < 0.001	*P* = 0.072	*P* = 0.207	*P* = 0.307	*P* = 0.117
I/II	0.859	0.860	0.892	0.924	0.955
III	0.722	0.788	0.850	0.958	0.932
Unknown	0.812	0.791	0.854	0.935	0.881
**Tumor Grade**	*P* = 0.009	*P* = 0.175	*P* = 0.131	*P* = 0.473	*P* = 0.375
I/II	0.945	0.834	0.974	0.944	0.942
III/IV	0.787	0.820	0.882	1	0.956
Unknown	0.794	0.830	0.855	0.922	0.928

* The value at year N from diagnosis was the CS for an additional year at year N-1.

**Figure 1:**
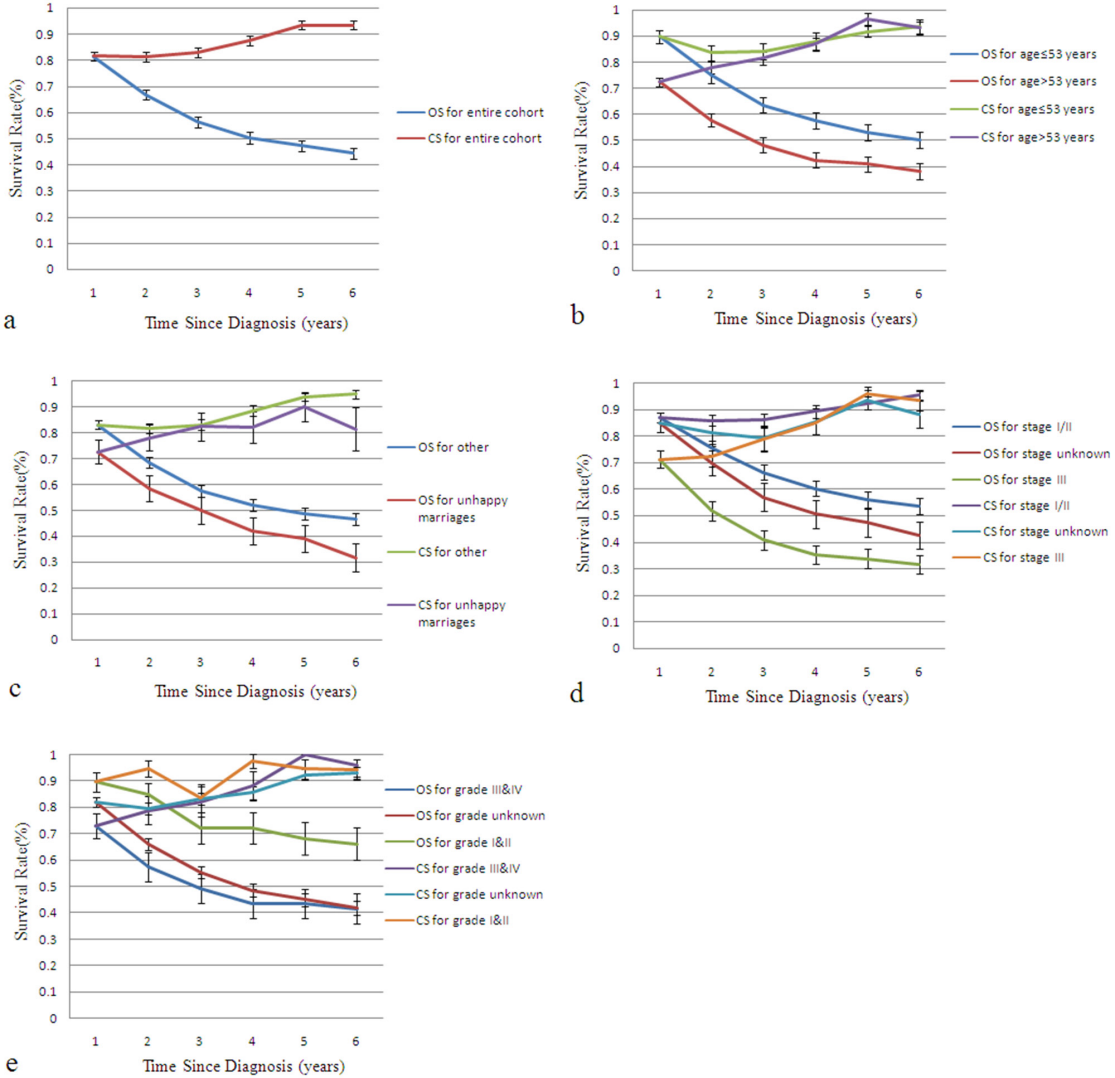
Kaplan-Meier curves for overall survival and conditional survival Kaplan-Meier curves for overall survival (OS) and 1-year conditional survival (CS) among the entire cohort of adrenal cortical carcinoma patients **a.** and patients segregated based on age **b.** marital status **c.** ENSAT stage **d.** and grade **e.**

CS estimates are more encouraging than static survival probabilities (Figure 1). Patients who had survived >24 months after their initial diagnosis had a better 1-year CS than those who had survived <24 months since diagnosis. Age, tumor stage, marital status and tumor grade also significantly affected CS. The gains in 1-year CS over time were more pronounced in older patients (>53 years), those with an unhappy marital status (divorced, separated or widowed), or stage III or high-grade disease. These differences in CS between different stage and grade groups decreased with time from diagnosis, or even disappeared.

## DISCUSSION

ACC is a rare malignancy that originates in the adrenal cortex and has a bimodal age distribution, with peaks in childhood and in the fourth to fifth decade of life [3]. Because ACC in children is more likely to be part of a rare hereditary syndrome [3], patients less than 18 years of age were excluded from our analysis to ensure we were focusing on the sporadic tumors that comprise most ACCs.

Surgery is the single most important intervention in the treatment of nonmetastatic ACC [5]. And even after recurrence or metastasis, surgery with the aim of tumor removal may be regarded as a potential treatment option [6, 7]. Icard *et al*. reported stage I, II and III 5-year survival rates after surgery to be 66%, 58% and 24%, respectively [8]. Ohwada *et al*. reported 5-year post-surgical survival rates of 20% and 40% for stages III and IV, respectively [9]. Using data from the SEER registry, Tran *et al*. found that the absence of cancer-directed surgery is a negative predictor of survival (hazard ratio, 3.341; confidence interval, 1.168–9.557) [10]. In the present study, we found that 5-year survival rates after surgery, and especially CS, justifies radical surgical resection in ACC patients, even those with advanced disease.

To our knowledge, this is the first study to address CS among patients with malignant ACC. Moreover, with 641 patients in the SEER registry (1988–2012), this is one of the largest studies of patients who have undergone surgery for non-metastatic malignant ACC. Compared to actual survival rates, CS probabilities appear to better reflect future survival of patients who have already survived for a given period of time.

Gains in 1-year CS over time are more pronounced in patients with poor risk factors than in those with favorable risk factors. For example, patients with advanced or poorly differentiated tumors have more pronounced gains than those with early-stage or well differentiated tumors. Kato *et al*. reported that the increase in median CS from 1 to 5 years after diagnosis is much slower in patients with prostate cancer (1.4-fold) than with lung cancer (13.0-fold) [11], reflecting the greater malignancy of lung cancer. Similarly, CS improved more in patients with colon [12, 13], ovarian [14] or rectal cancers [15] given poorer initial prognoses than in those with better prognoses. In the present study, CS improved over the first 5 years after diagnosis across all stages. For ENSAT stage III cancers, it improved from 72% at 1 year to 93% at 5 years, while for stage I/II cancers it steadily improved from 85% to 95% over the first 5 years. For most tumor types, CS at diagnosis is reportedly better in younger than older patients [16], and an unhappy marital status is associated with a higher risk of poor health [17]. CS thus improved more in older patients (>53 years) and those with unhappy marital status.

Although disease stage remains an important prognostic factor, earlier studies showed that between-stage differences in CS at diagnosis decrease with time from diagnosis, or even disappear [18, 19]. We found that for ACC between-stage differences in CS disappeared as follow-up became longer, and we observed a similar pattern for tumor grades. Although the effect of marital status on CS among ACC patients was not significant during the first 2 years after diagnosis, it became significant later during follow-up. The impact of age on CS was more complex. One year after diagnosis, older patients (>53 years) had worse 1-year CS, but this difference disappeared between the 2nd and 4th years after diagnosis. It then reappeared after 5 years, when older age became associated with a positive prognosis (see Table 4).

SEER data provide strong clinical insight into survival among patients in the U.S. with rare malignancies, including ACC. Although the overall quality of the SEER registry is impressive, it has several limitations [20]. The SEER registry does not always provide data concerning lymph node status. We treated the absence of data and negative lymph nodes as equivalent (negative). This is the main reason we did not assess the effect of lymphadenectomy, though it is an important prognostic factor in patients with ACC. Data concerning tumor markers, extent of surgery, completeness of resection and margin status were also unavailable. Although treatment with the adrenolytic drug mitotane or chemotherapy is important in advanced ACC cases and affects overall survival [21, 22], data concerning these drugs were not available in the SEER registry either. Finally, there is the unavoidable selection bias of all retrospective studies. Although these limitations may have played a role in the selection of available patients for this analysis, we remain confident that SEER audits medical records at participating institutions, enabling it to provide high quality data concerning ACC.

## MATERIALS AND METHODS

The SEER database was queried for patients over 18 years old diagnosed with ACC between 1988 and 2012. Based on the International Classification of Disease (ICD), patients with ACC were identified based on site (C74.0 or 74.9) and histologic subtype code (8370). Patients with distant metastases or other malignant tumors were excluded, as were those who had not undergone surgery for removal of their adrenal tumors.

Clinical stage was determined according to the ENSAT system, which is superior to the system of the Union for International Cancer Control (UICC; Table 1) [5, 23]. Stage I disease was defined as T1 N0 M0, stage II as T2 N0 M0, and stage III as T3 to 4 N0 M0 and T1 to 4 N1 M0. Tumor grading and differentiation were defined according to the ICD-Oncology-2 (ICD-O-2) in the SEER database [24]: well differentiated, Grade I; moderately differentiated, Grade II; poorly differentiated, Grade III; undifferentiated, anaplastic, Grade IV.

The subjects' relevant sociological, clinical and pathologic characteristics were described using simple summary statistics. The χ^2^ test and analysis of variance were used to analyze categorical and continuous variables, respectively. The Kaplan-Meier method was used for univariate analysis of prognostic factors, and the log-rank test was used to calculate statistical significance. Multivariate analysis was performed using a Cox proportional hazard model and forward stepwise method to determine predictors of survival.

Actual survival curves were generated using the Kaplan-Meier method. CS was defined as the probability of surviving for an additional year on the condition that a patient had already survived for a designated length of time [25]. The mathematical definition of CS can be expressed as follows: Let S(t) be the traditional actuarial life-table survival at time t. Conditional survival, CS(y/x), is the probability of surviving an additional y years, given that the patient has already survived x years. CS can then be expressed as: CS(y/x) = S(x+y)/S(x). For example, to compute the 1-year CS for patients who have survived 1 year, the 2-year survival is divided by the 1-year survival.

Analyses were performed using R software version 3.1.3 (R Development Core Team 2015) [26]. All tests of statistical significance were two-sided, and statistical significance was set at *P* < 0.05.
